# Efficacy and Safety of Recombinant Human Prourokinase in Acute Ischemic Stroke Within 4.5 h: A Systematic Review and Meta‐Analysis of Randomized Controlled Trials

**DOI:** 10.1002/brb3.70420

**Published:** 2025-03-13

**Authors:** Muhammad Hassan Waseem, Zain Ul Abideen, Aiman Waheed, Hafsa Arshad Azam Raja, Sanan Rasheed, Muhammad Mukhlis, Muhammad Abdullah Ali, Marium Khan, Umama Alam, Muhammad Fawad Tahir, Javed Iqbal, Ubaid Farooq, Sania Aimen

**Affiliations:** ^1^ Allama Iqbal Medical College Lahore Pakistan; ^2^ King Edward Medical University Lahore Pakistan; ^3^ Rawalpindi Medical University Rawalpindi Pakistan; ^4^ Ayub Medical College Abbottabad Pakistan; ^5^ Khyber Medical College Peshawar Pakistan; ^6^ Jinnah Sindh Medical University Karachi Pakistan; ^7^ HBS Medical and Dental College Islamabad Pakistan; ^8^ Hamad Medical Corp Doha Qatar; ^9^ Sheikh Zayed Medical College Rahim Yar Khan Pakistan; ^10^ Quetta Institute of Medical Sciences Quetta Pakistan

**Keywords:** alteplase | ischemic stroke | meta‐analysis | recombinant human prourokinase | systematic review

## Abstract

**Background:**

Acute ischemic stroke (AIS) requires timely thrombolysis to restore perfusion and minimize neurological damage. Recombinant human prourokinase (rhPro‐UK) has emerged as a promising alternative to alteplase, with potential efficacy and safety benefits within the critical 4.5‐h treatment window.

**Methods:**

Electronic databases, including PubMed, ScienceDirect, and Cochrane Central, were comprehensively searched from inception until December 2024. Risk ratios (RRs) with 95% confidence intervals were pooled for the dichotomous outcomes using a random effects model in Review Manager software. The heterogeneity among the included trials was evaluated using the *I* ^2^ statistics, and a sensitivity analysis was conducted to investigate the source of heterogeneity.

**Results:**

The final statistical analysis included 1179 participants in the rhPro‐UK and 1148 in the tPA group. Excellent functional outcome (modified Rankin Scale [mRS] 0‐1) (RR = 1.04, 95% CI: [0.98, 1.10]; *p* = 0.16) and good functional outcome (mRS 0‐2) (RR = 1.00, 95% CI: [0.96, 1.05]; *p* = 0.90; *I* ^2^ = 0%) were comparable between the two groups. There was also no significant difference in mortality and major neurological improvement. However, there was a trend toward a lower risk of symptomatic intracranial hemorrhage (sICH) in the rhPro‐UK group (RR = 0.53, 95% CI: [0.18, 1.59]; *p* = 0.26).

**Conclusion:**

rhPro‐UK demonstrated comparable efficacy to alteplase in achieving functional outcomes in AIS within 4.5 h, with no significant differences in mortality or neurological improvement. Although not statistically significant, a trend toward lower sICH risk with rhPro‐UK highlights its potential safety advantage. More high‐quality randomized clinical trials are required to confirm these findings.

## Graphical abstract

Recombinant human prourokinase demonstrates comparable efficacy and safety outcomes to alteplase in acute ischemic stroke patients treated within 4.5 h. These findings support its potential as an alternative thrombolytic agent.

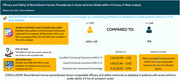

## 1 Introduction

Acute ischemic stroke (AIS) is a medical emergency indicated by the sudden disruption of blood flow to a part of the brain, leading to neuronal damage and loss of neurological function (Kuriakose and Xiao 2020). It is the leading cause of morbidity and mortality, prevailing in 68.16 million people worldwide, with an increasing trend emphasizing the critical need for effective and timely therapeutic interventions (Capirossi et al. 2023). The administration of thrombolytic agents within 4.5 h of symptom onset is pivotal in improving clinical outcomes by promoting reperfusion and minimizing cerebral infarction. Many thrombolytic agents, such as alteplase (tPA), tenecteplase (TNK), reteplase (rPA), urokinase (UK), recombinant human prourokinase (rhPro‐UK), streptokinase, and many others, have been considered for the therapeutic management of AIS. Since its approval, alteplase, a recombinant tissue plasminogen activator (rt‐PA), has been considered the most reliable agent for intravenous thrombolysis in AIS when administered in early4.5 h (Berge et al. 2021). However, it is not without limitations, such as the increased risk of intra‐cerebral hemorrhage, shorter half‐life, a narrow therapeutic window, and expense to utilize (Correa‐Paz et al. 2021). Its comparatively low clinical utilization rate is further influenced by its expensive cost and short treatment window (less than 4.5 h). Its application necessitates sophisticated imaging in later time periods (beyond 4.5 h).

Tenecteplase, a genetic variant of alteplase, offered superiority over alteplase in terms of functional outcomes and improvement in clinical symptoms. It can be administered as a single bolus, facilitating its clinical use (Rehman et al. 2023; Shen et al. 2023). Although alteplase's importance cannot be denied, its safety and efficacy remain comparable. Reteplase, a newer modified plasminogen activator, has been proven more efficacious than alteplase regarding functional and neurological outcomes (Li et al. 2024). However, its efficacy is comparable to alteplase in myocardial infarction. Its role in managing stroke has been less established until now. rhPro‐UK is a novel thrombolytic agent. This single‐chain zymogenic plasminogen activator specifically activates plasminogen on the fibrin surface, leading to targeted clot lysis and facilitating the breakdown of clots, obstructing blood flow to the brain. It is delivered intravenously and has demonstrated improved outcomes when administered within 4.5 h of symptom onset in AIS patients (Song et al. 2023b). rhPro‐UK is the synthetic form of this thrombolytic agent. It is less expensive and cost‐effective in lower–middle‐income countries (LMICs) than alteplase (Kharel et al. 2022).

There is an ongoing quest for the efficacy and safety profiles of novel agents to minimize the risk of hemorrhage, and rhPro‐UK has gained attention in recent clinical trials. This systematic review and meta‐analysis seek to rigorously evaluate the comparative effectiveness and safety of rhPro‐UK and alteplase in managing AIS within the critical 4.5‐h therapeutic window. By synthesizing data from multiple randomized controlled trials (RCTs), this study aims to provide a robust and comprehensive analysis of the available evidence, addressing critical gaps in current clinical practice and informing future therapeutic strategies.

## 2 Methods

This systematic review and meta‐analysis were carried out according to the standards provided by the Preferred Reporting Items for Systematic Reviews and Meta‐Analysis (PRISMA) guidelines. Before the review, the study protocol was registered in PROSPERO (Code: CRD42025634707).

### 2.1 Literature Search

A computerized search was run on PubMed, ScienceDirect, and Cochrane Central Registry from inception till December 2024 for studies comparing rhPro‐UK and alteplase in AIS patients using an extensive search strategy. For Medical Subject Heading (MeSH) terms or keywords, the terms (“Ischemic Stroke”) AND (“saruplase”) OR (“pro‐urokinase”) AND (“Tissue Plasminogen Activator”) OR (“Alteplase”) were merged. In addition, we thoroughly assessed the references of included studies and the relevant systematic reviews to reveal further eligible articles. The comprehensive search strategy can be found in Table S1.

### 2.2 Selection Criteria

The inclusion criteria were (1) study design: RCTs, (2) patient population: patients with AIS presenting with 4.5 h, (3) intervention: rhPro‐UK, and (4) comparator: alteplase (tPA).

The exclusion criteria included (1) animal studies, (2) in vivo studies, (3) non‐English articles, (4) articles with full text not retrieved, (5) studies without a comparator group, and (6) published letters, case reports, cohort studies, and narrative or systematic reviews.

### 2.3 Study Screening and Data Extraction

The articles retrieved from the detailed database search were imported to EndNote version 20. The duplicates were removed. Two reviewers screened the titles and abstracts of the remaining articles individually against pre‐determined eligibility criteria. A full‐text screening followed. Any disagreements were resolved by discussing them with a third author.

Data from the included articles were precisely extracted and filed using a Microsoft Excel sheet. Dichotomous outcomes were quantified as events and totals. Baseline characteristics reported were as follows: study ID, country, study design, sample size, mean age in years, male %, intervention, hypertension, diabetes, prior stroke, atrial fibrillation, drinking, current smoker, blood pressure, and baseline National Institutes of Health Stroke Scale (NIHSS) score. The primary and secondary outcomes of interest extracted were (1) excellent functional outcome (modified Rankin Scale [mRS] 0‐1) at 3 months, (2) good functional outcome (mRS 0‐2) at 3 months, (3) poor functional outcome (mRS 5‐6) at 3 months, (4) symptomatic intracranial hemorrhage (sICH), (5) mortality at 3 months, and (6) major neurological improvement within 72 h.

### 2.4 Study Endpoints

This study's primary and secondary endpoints were carefully selected to assess the clinical efficacy and safety of rhPro‐UK in patients with AIS treated within 4.5 h of symptom onset. The mRS was utilized to assess the functional neurological outcomes. The mRS is a widely used scale to measure the degree of disability or dependence in daily activities. Scores range from 0 to 6, where 0 indicates no symptoms and 6 indicates death. A lower score on the mRS indicates a better clinical outcome, with scores of 0–2 indicating mild to no disability and higher scores (3–6) indicating increasing levels of disability or death. A decrease in the mRS score indicates improvement in this endpoint, whereas worsening is indicated by an increase in the score. Major neurological improvement within 72 h was defined using the NIHSS. The NIHSS measures stroke severity, with scores ranging from 0 (no symptoms) to 42 (severe stroke). A higher score indicates worsening stroke severity and poorer outcomes, whereas a lower score indicates improved neurological function. Mortality was assessed as a dichotomous endpoint (alive vs. deceased) at the 3‐month follow‐up.

### 2.5 Bias Assessment

For the randomized control trials, we utilized the Cochrane Risk‐of‐Bias version‐2 (ROB‐2) tool to assess the risk of bias in the included studies (Higgins et al. 2011). This tool assesses the bias for five domains: (1) the bias stemming from the randomization process itself; (2) the bias due to deviation from the intended intervention; (3) the bias originated from missing outcome data; (4) the bias that is caused by measurement of outcome; (5) the bias because of the selection of reported results. The bias in the included studies was ranked as low, moderate, or some concern. Two authors independently reviewed bias in studies, and a senior author was contacted in case of discrepancies.

### 2.6 Statistical Analysis

The statistical analysis was performed using Review Manager version 5.4.1. Risk ratios (RRs) and 95% confidence intervals were pooled using the random effects model for dichotomous outcomes. A *p* value <0.05 was considered statistically significant. The results of the meta‐analysis were visually presented as forest plots. The heterogeneity between the included studies was evaluated using the *I* ^2^ statistics (Higgins and Green 2008). A leave‐one‐out sensitivity analysis was performed in case of high heterogeneity (*I* ^2^ > 50%). We also performed the GRADE assessment to determine the certainty of evidence. The publication bias was not assessed in this meta‐analysis due to a fewer number of studies.

## 3 Results

### 3.1 Search Results

**FIGURE 1:**
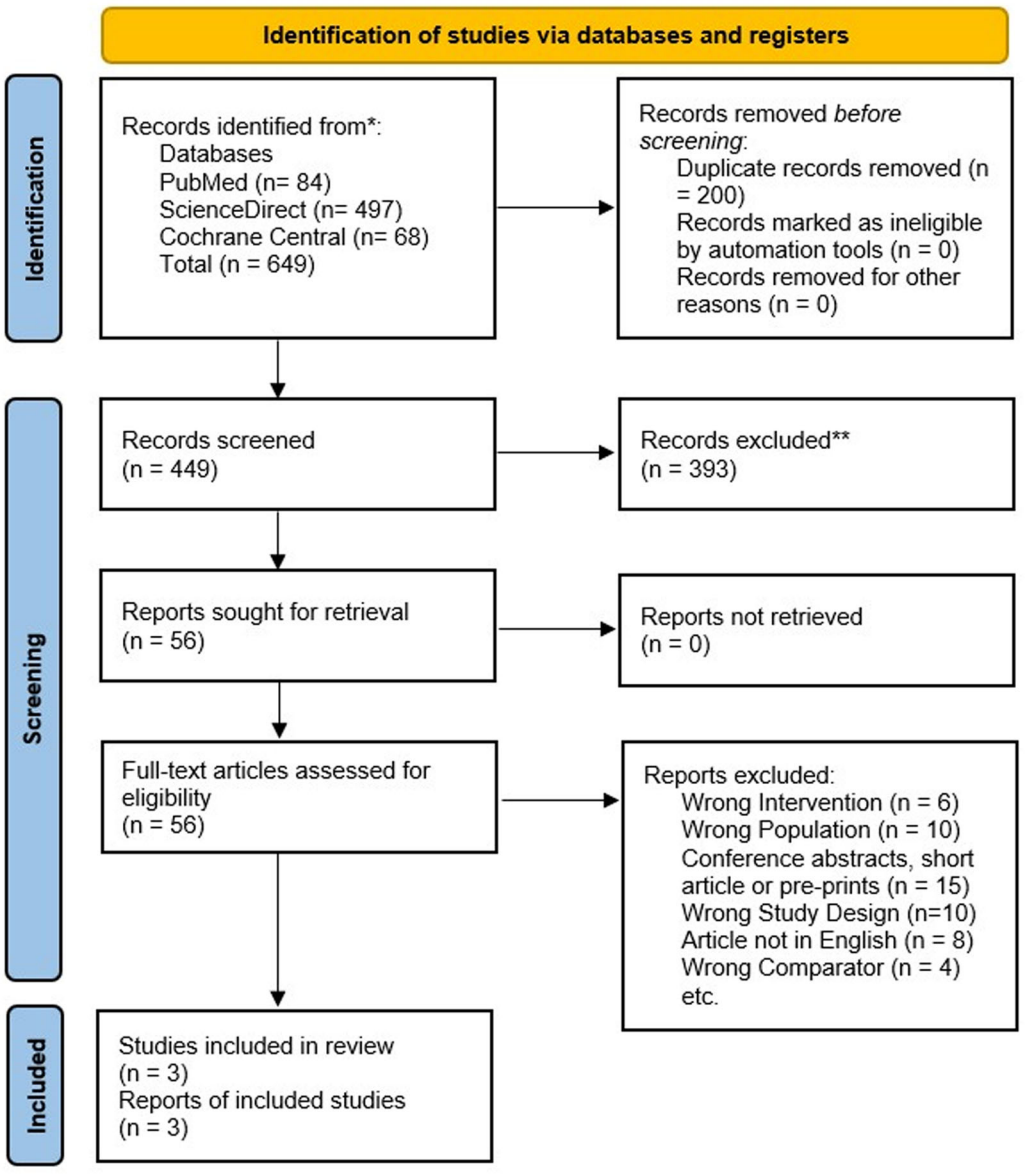
PRISMA diagram for study selection.

A total of 649 articles were retrieved by searching databases like PubMed, ScienceDirect, and Cochrane Library. After removing duplicates (*n* = 200), we were left with 449 articles, which passed through the title and abstract screening process, yielding 56 articles. These articles were then filtered through the full‐text screening, thus giving us a total of three studies to be included in the final quantitative analysis. The PRISMA flowchart explaining the study selection process thoroughly is depicted in Figure 1.

### 3.2 Characteristics of the Included Studies

This meta‐analysis pooled three studies, all RCTs, published between 2022 and 2024, covering 2320 patients. The sample size ranged from 112 to 1545, whereas the mean age ranged from 60.5 to 64.4 years. About 68% of participants had hypertension, 24.5% had diabetes, and 29.4% reported prior stroke. The reported NHSS score at baseline was 7.61 for rhPro‐UK and 7.5 for tPA. The details of the baseline characteristics of the included studies are given in Table 1.

**TABLE 1:** Characteristics of the included studies.

Study ID	Country	Study design	Sample size (*n*)	Mean age in years (SD)	Male (%)	Intervention	Hypertension (%)	Diabetes (%)	Prior stroke (%)	Atrial fibrillation (%)	Drinking (%)	Current smoker (%)	Blood pressure, mean (SD), mm HG		Baseline NIHSS score, mean (SD)	
													Systolic	Diastolic	rhPro‐UK	tPA
PROST‐2 2024	China	RCT	1545	64.4 (10.7)	66.4	rhPro‐UK (35 mg) tPA (0.90 mg/kg)	76.9	26.5	33.1	7.3	NR	NR	NR	NR	7 (2.97)	7.33 (2.23)
Song 2022	China	RCT	112	60.5 (9.7)	65.1	rhPro‐UK (35 mg and 50 mg) tPA (0.90 mg/kg)	66.9	25	29.4	NR	9.8	35.7	152.88 (18.74)	88.17 (12.42)	8.21 (3.66)	8.08 (4.49)
Song 2023a	China	RCT	663	61 (10.2)	75.7	rhPro‐UK (35 mg) tPA (0.90 mg/kg)	62.4	22	25.7	NR	6.9	39.3	150.79 (21.63)	86.91 (12.66)	7.62 (3.71)	7.29 (3.40)

Abbreviations: NIHSS, National Institutes of Health Stroke Scale; RCT, randomized controlled trial; rhPro‐UK, recombinant human prourokinase; SD, standard deviation; tPA, alteplase.

### 3.3 Risk of Bias Assessment and GRADE Assessment

**FIGURE 2:**
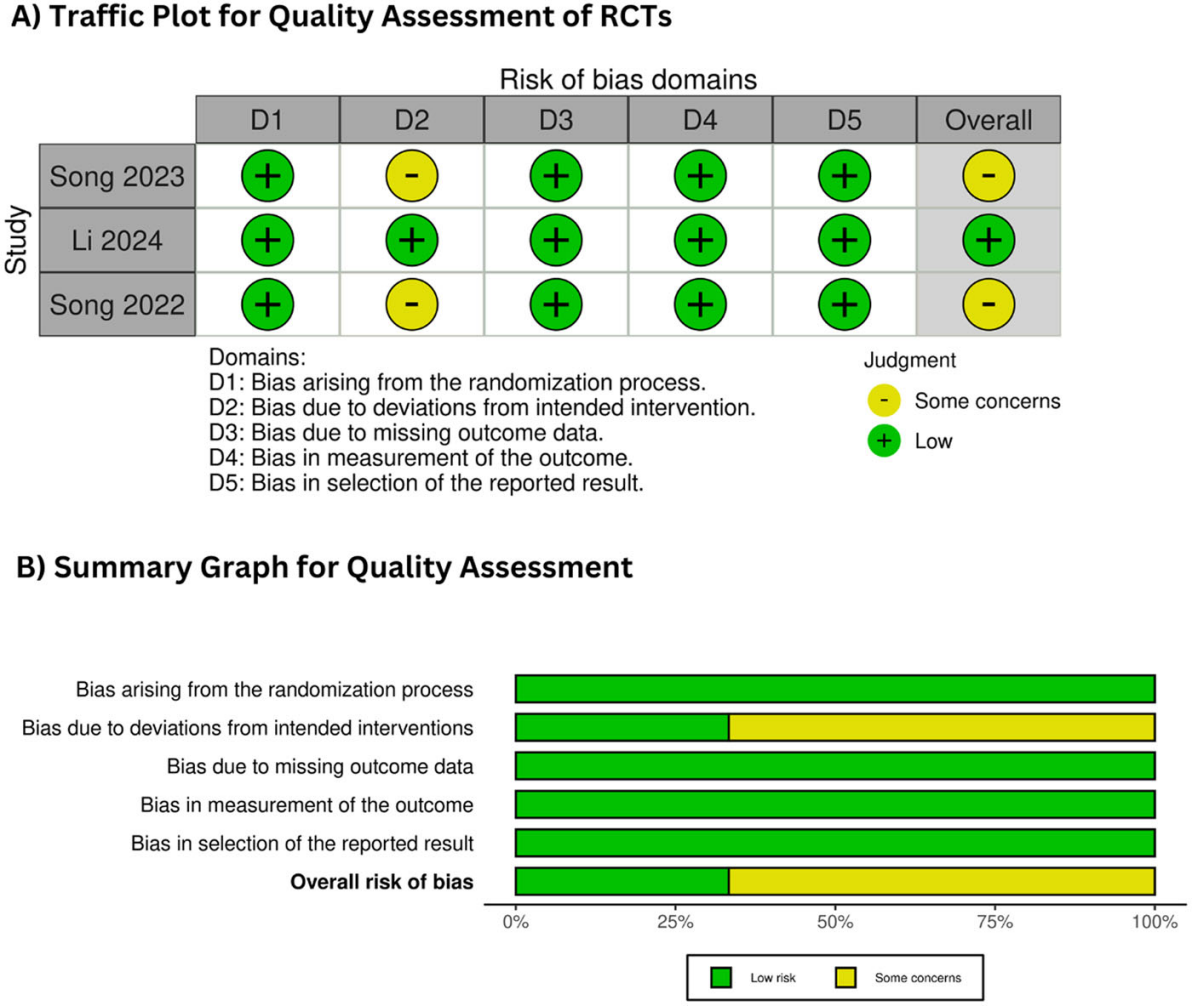
(A) Traffic plot for quality assessment of RCTs; (B) summary graph for quality assessment. RCT, randomized controlled trial.

The quality of the RCTs was assessed through the Cochrane RoB 2.0 tool (Higgins et al. 2011). One study indicated a low risk of bias (Li et al. 2025), whereas two studies (Song et al. 2022, 2023a) presented an uncertain risk. Details of the quality assessment of the RCTs are given in Figure 2. The GRADE assessment was performed to determine the certainty of evidence. The evidence demonstrated high certainty regarding excellent and good functional outcomes, whereas sICH and poor functional outcomes exhibited low certainty. The details are provided in Table 2.

**TABLE 2:** GRADE assessment.

Prourokinase compared to alteplase for acute ischemic stroke					
**Patient or population**: Acute ischemic stroke **Setting**: **Intervention**: Prourokinase **Comparison**: Alteplase					
Outcomes	Anticipated absolute effects^*^ (95% CI)		Relative effect (95% CI)	No. of participants (studies)	Certainty of the evidence (GRADE)
	**Risk with alteplase**	**Risk with prourokinase**			
Excellent functional outcome (mRS 0‐1)	669 per 1000	**696 per 1000** (656–736)	**RR 1.04** (0.98–1.10)	2327 (3 RCTs)	⨁⨁⨁⨁ High
Good functional outcome (mRS 0‐2)	777 per 1000	**777 per 1000** (746–816)	**RR 1.00** (0.96–1.05)	2327 (3 RCTs)	⨁⨁⨁⨁ High
Major neurological improvement within 72 h	461 per 1000	**484 per 1000** (442–525)	**RR 1.05** (0.96–1.14)	2327 (3 RCTs)	⨁⨁⨁◯ Moderate ^a^
Mortality at 90 days	35 per 1000	**37 per 1000** (23–62)	**RR 1.07** (0.65–1.78)	2321 (3 RCTs)	⨁⨁⨁◯ Moderate^b^
Symptomatic intracranial hemorrhage	14 per 1000	**7 per 1000** (3–22)	**RR 0.53** (0.18–1.59)	2321 (3 RCTs)	⨁⨁◯◯ Low^a,b^
Poor functional outcome (mRS 5‐6)	44 per 1000	**67 per 1000** (28–162)	**RR 1.50** (0.62–3.64)	2327 (3 RCTs)	⨁⨁◯◯ Low^a,b^

*Note*: “a”—Individual trials showed varying effect estimates; “b”—small sample size leading to wider confidence intervals.

Abbreviations: CI, confidence interval; mRS, modified Rankin Scale; RCT, randomized controlled trial; RR, risk ratio.

*The risk in the intervention group (and its 95% confidence interval) is based on the assumed risk in the comparison group and the **relative effect** of the intervention (and its 95% CI).

### 3.4 Outcomes

#### 3.4.1 Excellent Functional Outcome (mRS 0‐1)

**FIGURE 3:**
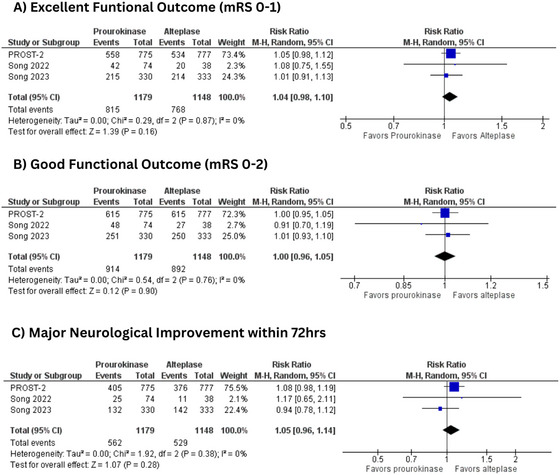
(A) Excellent functional outcome (mRS 0‐1); (B) good functional outcome (mRS 0‐2); (C) major neurological improvement within 72 h. mRS, modified Rankin Scale.

Three studies (Li et al. 2025; Song et al. 2022, 2023a) with 2327 patients reported an excellent functional outcome (mRS 0‐1) (rhPro‐UK = 1179 vs. tPA = 1148). The two thrombolytic agents were comparable in terms of excellent functional outcome (RR = 1.04, 95% CI: [0.98, 1.10]; *p* = 0.16). Heterogeneity was low between the studies (*I* ^2^ = 0%) (Figure 3A).

#### 3.4.2 Good Functional Outcome (mRS 0‐2)

Three trials with 2327 patients reported good functional outcomes (mRS 0‐2) (rhPro‐UK = 1179 vs. tPA = 1148). The two groups showed no statistically significant difference regarding this outcome (RR = 1.00, 95% CI: [0.96, 1.05]; *p* = 0.90; *I* ^2^ = 0%) (Figure 3B).

#### 3.4.3 Major Neurological Improvement Within 72 h

Three trials encompassing 2321 patients analyzed major neurological improvement within 72 h (rhPro‐UK = 1179; tPA = 1148) (Li et al. 2025; Song et al. 2022, 2023a). There was no significant difference between prourokinase and alteplase regarding major neurological improvement within 72 h (RR = 1.05, 95% CI: [0.96, 1.14]; *p* = 0.28). The heterogeneity between the studies was low (*I* ^2^ = 0%) (Figure 3C).

#### 3.4.4 All‐Cause Mortality

**FIGURE 4:**
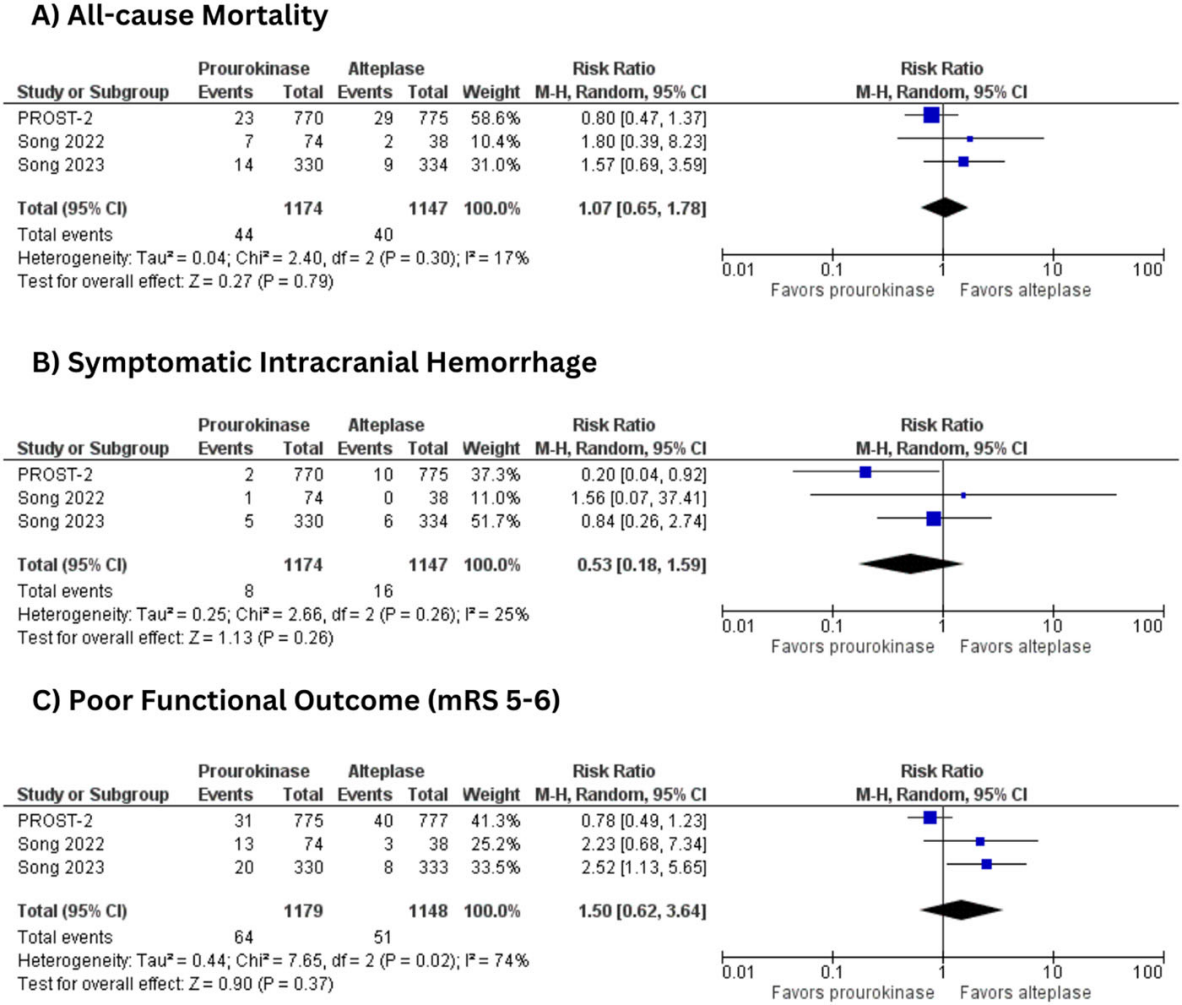
(A) All‐cause mortality; (B) symptomatic intracranial hemorrhage; (C) poor functional outcome (mRS 5‐6). mRS, modified Rankin Scale.

Mortality at 3 months was examined in three included trials involving 2321 patients (rhPro‐UK = 1174 vs. 1147). The mortality risk was comparable between the two thrombolytic agents (RR = 1.07, 95% CI: [0.65, 1.78]; *p* = 0.79). Heterogeneity between the studies was low (*I* ^2^ = 17%) (Figure 4A).

#### 3.4.5 Symptomatic Intracranial Hemorrhage

sICH was reported in three trials (Li et al. 2025; Song et al. 2022, 2023a) with 2321 patients (rhPro‐UK = 1174 vs. tPA = 1147). The prourokinase group showed a lower risk of sICH, but the results were statistically non‐significant (RR = 0.53, 95% CI: [0.18, 1.59]; *p* = 0.26). Heterogeneity between the studies was low (*I* ^2^ = 25%) (Figure 4B).

#### 3.4.6 Poor Functional Outcome (mRS 5‐6)

Three trials involving 2327 patients reported poor functional outcomes (mRS 5‐6) (rhPro‐UK = 1179 vs. tPA = 1148). The two groups had no significant difference (RR = 1.50, 95% CI: [0.62, 3.64]; *p* = 0.37; *I* ^2^ = 74%) (Figure 4C).

#### 3.4.7 Sensitivity Analysis

To address the robustness of our findings, we performed a leave‐one‐out sensitivity analysis. This analysis involved sequentially removing each study from the analysis to evaluate whether any individual study disproportionately impacted the overall effect size. The results showed that excluding any single study did not significantly alter the overall outcomes, indicating that the findings of our meta‐analysis are robust and not driven by any one study.

## 4 Discussion

AIS continues to be one of the leading causes of morbidity and mortality in the globe, and intravenous thrombolysis with tissue plasminogen activator (tPA, also known as alteplase) serves as the mainstay of therapy. However, alteplase is accompanied by limitations, such as a restricted treatment window (within 4.5 h of symptom onset), sICH risk, and logistical challenges connected to its administration and management. The search for alternatives to alteplase, which are equally effective, safer, and more accessible, has led to research into rhPro‐UK. This meta‐analysis compares rhPro‐UK to alteplase in terms of efficacy and safety outcomes.

Our study found that rhPro‐UK demonstrated comparable efficacy to alteplase in achieving excellent and good functional outcomes in patients with AIS. This finding aligns with the results from Liu et al. (2021), who reported similar functional outcomes with rhPro‐UK and alteplase. Zhao et al. (2018) also observed comparable efficacy in rhPro‐UK when used alongside mechanical thrombectomy for moderate‐to‐severe cerebral infarction. Furthermore, both studies highlighted that rhPro‐UK could offer similar benefits as alteplase in achieving functional independence and improving patient outcomes, thus supporting its potential as a viable alternative for thrombolytic therapy.

Our study's analysis of mortality at 3 months showed comparable results between rhPro‐UK and alteplase. This is consistent with the findings of Li et al. (2025), who reported similar mortality rates between rhPro‐UK and alteplase in their analysis of AIS patients. Additionally, our results align with those of Zhao et al. (2018), where no significant difference in mortality was observed between the two agents.

Regarding major neurological improvement within 72 h, our study revealed no significant difference between rhPro‐UK and alteplase. This supports the findings of another study (Zhao et al. 2018), which also reported no significant difference in neurological improvement between the two agents. The low heterogeneity further validates the robustness of our findings and aligns with the results from Liu et al. (2021), where no significant discrepancies were reported in terms of neurological recovery between rhPro‐UK and alteplase. These consistent results strengthen the argument for rhPro‐UK as a comparable treatment option to alteplase regarding neurological recovery post‐thrombolysis.

In our study, the safety profile of rhPro‐UK was comparable to that of alteplase, with a slightly lower incidence of sICH. Although the difference in bleeding complications between rhPro‐UK and alteplase was not statistically significant, the reduced trend in bleeding events with rhPro‐UK suggests a potential advantage in terms of safety, particularly for high‐risk patients. Our results also showed no significant difference in major bleeding events. Similar findings were reported by Liu et al. (2021), who found no significant difference in the incidence of bleeding complications between rhPro‐UK and alteplase. However, the potential for reduced hemorrhagic risk with rhPro‐UK requires further exploration in more extensive, multicenter trials, especially considering that even small reductions in sICH risk could have significant clinical implications for patient outcomes.

Furthermore, rhPro‐UK's safer profile may render it a more appealing option for patients at heightened risk of hemorrhagic complications, such as those with pre‐existing conditions like hypertension or a history of stroke. Our study found no significant difference between rhPro‐UK and alteplase regarding poor functional outcomes. This aligns with the results of Li et al. (2025), who found no statistically significant difference in poor functional outcomes between rhPro‐UK and alteplase in their Phase 3 trial. Similarly, van der Ende et al. (2022) did not observe a significant difference in poor functional outcomes between the two agents in their study involving dual thrombolytic therapy.

One of the advantages of rhPro‐UK over alteplase is the potential for faster administration. Several studies have looked into the timing of thrombolysis because early reperfusion is critical for reducing neurological deficits. Song et al. (2023b) showed that rhPro‐UK was safe and effective when administered within a window of 4.5–6 h post‐stroke. In our study, rhPro‐UK demonstrated a significantly faster time to thrombolysis (85 min) compared to alteplase (120 min), which could improve early reperfusion in AIS. Faster administration of rhPro‐UK may help reduce treatment delays, offering a potential advantage over alteplase in timely stroke management. The faster treatment time for rhPro‐UK might help increase the number of patients who can benefit from timely thrombolysis, particularly in settings with logistical constraints.

The findings of this meta‐analysis carry significant clinical implications, particularly in expanding thrombolytic options for AIS. The comparable efficacy and safety profiles of rhPro‐UK to alteplase, coupled with its trend toward reduced sICH, position rhPro‐UK as a viable alternative, especially in resource‐limited settings where cost and logistical barriers hinder alteplase use. Future research should prioritize large‐scale, multicenter RCTs to validate these findings across diverse ethnic and geographic populations. Investigations into rhPro‐UK's efficacy in extended time windows (beyond 4.5 h) or in combination with mechanical thrombectomy could further broaden its clinical utility. Additionally, long‐term follow‐up studies are essential to assess outcomes like recurrent stroke rates, quality of life, and disability metrics, which remain understudied. Finally, cost‐effectiveness analyses in real‐world settings could strengthen advocacy for rhPro‐UK's adoption in national stroke guidelines, particularly in LMICs where economic constraints limit access to current standards of care.

### 4.1 Limitations

Despite our study's promising results, the current literature has limitations. For instance, although we have demonstrated comparable efficacy between rhPro‐UK and alteplase regarding functional outcomes, further large‐scale multicenter trials are required to confirm these findings across diverse populations and clinical settings. Moreover, the long‐term outcomes of rhPro‐UK, including recurrent stroke rates and post‐stroke disability, remain underexplored. Del Zoppo et al. (1998) found some variations in efficacy between different thrombolytic agents, indicating the need for further investigation. Future trials should also compare long‐term quality of life and disability outcomes to assess rhPro‐UK's full potential.

## 5 Conclusion

In conclusion, rhPro‐UK demonstrates promising results comparable to alteplase for treating AIS, with advantages in a potentially safer bleeding profile. These findings support rhPro‐UK as a potential alternative thrombolytic agent to alteplase, particularly in resource‐limited settings. However, further trials, including more extensive multicenter studies and long‐term follow‐ups, are required to establish its full clinical value.

## Author Contributions

**Muhammad Hassan Waseem**: conceptualization, writing–original draft, supervision. **Zain Ul Abideen**: writing–original draft, methodology, software. **Aiman Waheed**: investigation, writing–review and editing, visualization. **Hafsa Arshad Azam Raja**: project administration, formal analysis, software. **Sanan Rasheed**: data curation, resources, writing–original draft. **Muhammad Mukhlis**: methodology, data curation. **Muhammad Abdullah Ali**: project administration, writing–original draft. **Marium Khan**: formal analysis, writing–original draft. **Umama Alam**: software, investigation. **Muhammad Fawad Tahir**: data curation, writing–original draft. **Javed Iqbal**: writing–review and editing, validation, funding acquisition. **Ubaid Farooq**: writing–review and editing, supervision. **Sania Aimen**: writing–review and editing, validation.

## Conflicts of Interest

The authors declare no conflicts of interest.

## Ethics Statement

The authors have nothing to report.

### Peer Review

The peer review history for this article is available at https://publons.com/publon/10.1002/brb3.70420

## Supporting information



Table S1 Detailed search strategy.

## Data Availability

The data that support the findings of this study are available on request from the corresponding author. The data are not publicly available due to privacy or ethical restrictions.

## References

[brb370420-bib-0001] Berge, E. , W. Whiteley , H. Audebert , et al. 2021. “European Stroke Organisation (ESO) Guidelines on Intravenous Thrombolysis for Acute Ischaemic Stroke.” European Stroke Journal 6: I–LXII. 10.1177/2396987321989865. PMC7995316 33817340

[brb370420-bib-0002] Capirossi, C. , A. Laiso , L. Renieri , F. Capasso , and N. Limbucci . 2023. “Epidemiology, Organization, Diagnosis and Treatment of Acute Ischemic Stroke.” European Journal of Radiology Open 11: 100527. 10.1016/J.EJRO.2023.100527. 37860148 PMC10582298

[brb370420-bib-0003] Correa‐Paz, C. , A. da Silva‐Candal , E. Polo , et al. 2021. “New Approaches in Nanomedicine for Ischemic Stroke.” Pharmaceutics 13: 757. 10.3390/PHARMACEUTICS13050757. 34065179 PMC8161190

[brb370420-bib-0004] Del Zoppo, G. J. , R. T. Higashida , A. J. Furlan , M. S. Pessin , H. A. Rowley , and M. Gent . 1998. “PROACT: A Phase II Randomized Trial of Recombinant Pro‐Urokinase by Direct Arterial Delivery in Acute Middle Cerebral Artery Stroke.” PROACT Investigators. Prolyse in Acute Cerebral Thromboembolism, Stroke 29: 4–11. 10.1161/01.STR.29.1.4. 9445320

[brb370420-bib-0005] Higgins, J. P. , and S. Green . 2008. Cochrane Handbook for Systematic Reviews of Interventions. Cochrane Collaboration. 10.1002/9780470712184.

[brb370420-bib-0006] Higgins, J. P. T. , D. G. Altman , P. C. Gøtzsche , et al. 2011. “The Cochrane Collaboration's Tool for Assessing Risk of Bias in Randomised Trials.” BMJ (Online) 343: d5928. 10.1136/BMJ.D5928. PMC3196245 22008217

[brb370420-bib-0007] Kharel, S. , G. Nepal , P. R. Joshi , J. K. Yadav , and T. M. Shrestha . 2022. “Safety and Efficacy of Low‐Cost Alternative Urokinase in Acute Ischemic Stroke: A Systematic Review and Meta‐Analysis.” Journal of Clinical Neuroscience 106: 103–109. 10.1016/J.JOCN.2022.09.015. 36274296

[brb370420-bib-0008] Kuriakose, D. , and Z. Xiao . 2020. “Pathophysiology and Treatment of Stroke: Present Status and Future Perspectives.” International Journal of Molecular Sciences 21: 1–24. 10.3390/IJMS21207609. PMC7589849 33076218

[brb370420-bib-0009] Leppert, M. H. , J. D. Campbell , J. R. Simpson , and J. F. Burke . 2015. “Cost‐Effectiveness of Intra‐Arterial Treatment as an Adjunct to Intravenous Tissue‐Type Plasminogen Activator for Acute Ischemic Stroke.” Stroke; A Journal of Cerebral Circulation 46: 1870–1876. 10.1161/STROKEAHA.115.009779. PMC4480156 26012639

[brb370420-bib-0010] Li, S. , H. Q. Gu , B. Feng , et al. 2025. “Safety and Efficacy of Intravenous Recombinant Human Prourokinase for Acute Ischaemic Stroke Within 4·5 h After Stroke Onset (PROST‐2): A Phase 3, Open‐Label, Non‐Inferiority, Randomised Controlled Trial.” Lancet Neurology 24: 33–41. 10.1016/S1474-4422(24)00436-8. 39617030

[brb370420-bib-0011] Li, S. , H.‐Q. Gu , H. Li , et al. 2024. “Reteplase Versus Alteplase for Acute Ischemic Stroke.” New England Journal of Medicine 390: 2264–2273. 10.1056/NEJMOA2400314. 38884332

[brb370420-bib-0012] Liu, Y. , Y. Yang , Y. Li , and X. Peng . 2021. “Comparison of Efficacy and Safety of Recombinant Human Prourokinase and Alteplase in the Treatment of STEMI and Analysis of Influencing Factors of Efficacy.” Evidence‐Based Complementary and Alternative Medicine 2021: 6702965. 10.1155/2021/6702965. 34531919 PMC8440075

[brb370420-bib-0013] Rehman, A. U. , A. Mohsin , H. A. Cheema , et al. 2023. “Comparative Efficacy and Safety of Tenecteplase and Alteplase in Acute Ischemic Stroke: A Pairwise and Network Meta‐Analysis of Randomized Controlled Trials.” Journal of the Neurological Sciences 445: 120537. 10.1016/J.JNS.2022.120537. 36630803

[brb370420-bib-0014] Shen, Z. , N. Bao , M. Tang , et al. 2023. “Tenecteplase vs. Alteplase for Intravenous Thrombolytic Therapy of Acute Ischemic Stroke: A Systematic Review and Meta‐Analysis.” Neurology and Therapy 12: 1553–1572. 10.1007/S40120-023-00530-4. 37552459 PMC10444744

[brb370420-bib-0015] Song, H. , Y. Wang , Q. Ma , et al. 2022. “Efficacy and Safety of Recombinant Human Prourokinase in Acute Ischemic Stroke: A Phase IIa Randomized Clinical Trial.” Translational Stroke Research 13: 995–1004. 10.1007/s12975-022-01012-9. 35505174

[brb370420-bib-0016] Song, H. , Y. Wang , Q. Ma , et al. 2023a. “Efficacy and Safety of Recombinant Human Prourokinase in the Treatment of Acute Ischemic Stroke within 4.5 Hours of Stroke Onset: A Phase 3 Randomized Clinical Trial.” JAMA Network Open 6: e2325415. 10.1001/jamanetworkopen.2023.25415. 37490291 PMC10370258

[brb370420-bib-0017] Song, H. , Y. Wang , Q. Ma , et al. 2023b. “Thrombolysis With Recombinant Human Prourokinase 4.5–6 h after Acute Ischemic Stroke: A Phase IIa, Randomized, and Open‐Label Multicenter Clinical Trial.” CNS Drugs 38: 67. 10.1007/S40263-023-01051-2. 38030867 PMC10811005

[brb370420-bib-0018] van der Ende, N. A. M. , B. Roozenbeek , L. E. M. Smagge , et al. 2022. “Dual Thrombolytic Therapy With Mutant Pro‐Urokinase and Small Bolus Alteplase for Ischemic Stroke (DUMAS): Study Protocol for a Multicenter Randomized Controlled Phase II Trial.” Trials 23: 641. 10.1186/S13063-022-06596-Z. 35945566 PMC9361639

[brb370420-bib-0019] Zhao, Q. S. , W. Li , D. Li , et al. 2018. “Clinical Treatment Efficiency of Mechanical Thrombectomy Combined With rhPro‐UK Thrombolysis for Acute Moderate/Severe Cerebral Infarction.” European Review for Medical and Pharmacological Sciences 22: 5740–5746. 10.26355/EURREV_201809_15842. 30229852

