# Supplementary material for: Efficacy and Safety of Recombinant Human Prourokinase in Acute Ischemic Stroke Within 4.5 h: A Systematic Review and Meta‐Analysis of Randomized Controlled Trials
Source: Brain Behav. 2025 Mar 13;15(3):e70420. doi: 10.1002/brb3.70420 (PMC11905063; doi:10.1002/brb3.70420)
Supplement: Supplementary file 1 — Table S1 Detailed search strategy. [file BRB3-15-e70420-s001.docx]

| Supplementary Table S1: Detailed search strategy | | |
| --- | --- | --- |
| Database | Search string | Results |
| PubMed | ((("recombinant"[All Fields] OR "recombinants"[All Fields] OR "recombinate"[All Fields] OR "recombinated"[All Fields] OR "recombinates"[All Fields] OR "recombination, genetic"[MeSH Terms] OR ("recombination"[All Fields] AND "genetic"[All Fields]) OR "genetic recombination"[All Fields] OR "recombination"[All Fields] OR "recombinations"[All Fields] OR "recombinational"[All Fields] OR "recombinative"[All Fields] OR "recombine"[All Fields] OR "recombined"[All Fields] OR "recombineered"[All Fields] OR "recombineering"[All Fields] OR "recombines"[All Fields] OR "recombining"[All Fields]) AND ("human s"[All Fields] OR "humans"[MeSH Terms] OR "humans"[All Fields] OR "human"[All Fields]) AND "prourokinase"[All Fields]) OR "prourokinase"[All Fields] OR "RhPRO-UK"[All Fields]) AND ("stroke"[MeSH Terms] OR "stroke"[All Fields] OR "strokes"[All Fields] OR "stroke s"[All Fields] OR ("ischemic stroke"[MeSH Terms] OR ("ischemic"[All Fields] AND "stroke"[All Fields]) OR "ischemic stroke"[All Fields]) OR ("ischemic stroke"[MeSH Terms] OR ("ischemic"[All Fields] AND "stroke"[All Fields]) OR "ischemic stroke"[All Fields] OR ("acute"[All Fields] AND "ischemic"[All Fields] AND "stroke"[All Fields]) OR "acute ischemic stroke"[All Fields])) | 84 |
| ScienceDirect | (recombinant human prourokinase OR prourokinase OR RhPRO-UK) AND (stroke OR ischemic stroke OR acute Ischemic stroke) | 497 |
| Cochrane Central | (recombinant human prourokinase OR prourokinase OR RhPRO-UK) AND (stroke OR ischemic stroke OR acute Ischemic stroke) | 68 |
